# Immunological assessment of SARS-CoV-2 infection in pregnancy from diagnosis to delivery: A multicentre prospective study

**DOI:** 10.1371/journal.pone.0253090

**Published:** 2021-09-20

**Authors:** Kate Glennon, Jennifer Donnelly, Susan Knowles, Fionnuala M. McAuliffe, Alma O’Reilly, Siobhan Corcoran, Jennifer Walsh, Roger McMorrow, Tess Higgins, Lucy Bolger, Susan Clinton, Sarah O’Riordan, Alexander Start, Doireann Roche, Helena Bartels, Ciara Malone, Karl McAuley, Anthony McDermott, Rosanna Inzitari, Colm P. F. O’Donnell, Fergal Malone, Shane Higgins, Cillian De Gascun, Peter Doran, Donal J. Brennan

**Affiliations:** 1 UCD School of Medicine, National Maternity Hospital, Dublin, Ireland; 2 RCSI School of Medicine, Rotunda Hospital, Dublin, Ireland; 3 Department of Microbiology, National Maternity Hospital, Dublin, Ireland; 4 UCD Perinatal Research Centre, School of Medicine, University College Dublin, National Maternity Hospital, Dublin, Ireland; 5 National Maternity Hospital, Dublin, Ireland; 6 Rotunda Hospital, Dublin, Ireland; 7 Clinical Research Centre, UCD School of Medicine, St Vincent’s University Hospital, Dublin, Ireland; 8 Neonatal Unit, UCD School of Medicine National Maternity Hospital, Dublin, Ireland; 9 National Virus Reference Laboratory, University College Dublin, Dublin, Ireland; 10 Systems Biology Ireland, UCD School of Medicine, Dublin, Ireland; Centers for Disease Control and Prevention, UNITED STATES

## Abstract

**Background:**

Background Population-based data on SARS-CoV-2 infection in pregnancy and assessment of passive immunity to the neonate, is lacking. We profiled the maternal and fetal response using a combination of viral RNA from naso-pharyngeal swabs and serological assessment of antibodies against SARS-CoV-2.

**Methods:**

This multicentre prospective observational study was conducted between March 24th and August 31st 2020. Two independent cohorts were established, a symptomatic SARS-CoV-2 cohort and a cohort of asymptomatic pregnant women attending two of the largest maternity hospitals in Europe. Symptomatic women were invited to provide a serum sample to assess antibody responses. Asymptomatic pregnant women provided a nasopharyngeal swab and serum sample. RT-PCR for viral RNA was performed using the Cobas SARS-CoV-2 6800 platform (Roche). Umbilical cord bloods were obtained at delivery. Maternal and fetal serological response was measured using both the Elecsys® Anti-SARS-CoV-2 immunoassay (Roche), Abbott SARS-CoV-2 IgG Assay and the IgM Architect assay. Informed written consent was obtained from all participants.

**Results:**

Ten of twenty three symptomatic women had SARS-CoV-2 RNA detected on nasopharyngeal swabs. Five (5/23, 21.7%) demonstrated serological evidence of anti-SARS-CoV-2 IgG antibodies and seven (30.4%, 7/23) were positive for IgM antibodies. In the asymptomatic cohort, the prevalence of SARS-CoV-2 infection in RNA was 0.16% (1/608). IgG SARS-CoV-2 antibodies were detected in 1·67% (10/598, 95% CI 0·8%-3·1%) and IgM in 3·51% (21/598, 95% CI 2·3–5·5%). Nine women had repeat testing post the baseline test. Four (4/9, 44%) remained IgM positive and one remained IgG positive. 3 IgG anti-SARS-CoV-2 antibodies were detectable in cord bloods from babies born to five seropositive women who delivered during the study. The mean gestation at serological test was 34 weeks. The mean time between maternal serologic positivity and detection in umbilical cord samples was 28 days.

**Conclusion:**

Using two independent serological assays, we present a comprehensive illustration of the antibody response to SARS-CoV-2 in pregnancy, and show a low prevalence of asymptomatic SARS-CoV2. Transplacental migration of anti-SARS-CoV-2 antibodies was identified in cord blood of women who demonstrated antenatal anti-SARS-CoV-2 antibodies, raising the possibility of passive immunity.

## Introduction

Despite swift advances in our understanding of the SARS-CoV-2 virus, much remains to be understood regarding the timing, nature and persistence of both the humoral and cellular human response. Confirmation of an antibody response in pregnant women can direct resources in maternal services but also in the management of neonates during future surges in a similar fashion that current antenatal influenza and pertussis vaccination schedules utilise the transplacental migration of antibodies to enhance the neonatal immune system [1]. In this study, we present a comprehensive profile of the temporal serological response in pregnant women and document the presence of transplacental antibodies to SARS-CoV-2.

Maternal IgG antibodies travelling across the placenta provide vital immunity to the newborn and have been demonstrated in infants for infections such as tetanus and human papillomavirus (HPV) [2]. To date, the evidence is sparse surrounding transplacental passage of SARS-CoV-2. Initially, at the outset of the pandemic, strict measures were adopted to reduce the risk of vertical transmission to the neonate, including isolation of babies from SARS-CoV-2 positive mothers [3]. Antibodies have been demonstrated in the blood of neonates born to positive mothers when tested at birth [4, 5] and evidence of maternal antibodies to SARS-CoV-2 within cord bloods is evident [6, 7]. Further confirmation of transplacental migration of maternal anti-SARS-CoV-2 antibodies in umbilical cord blood could suggest the possibility of passive immunity and could even direct vaccination protocols in pregnant women.

Determining the seroprevalence of SARS-CoV-2 has largely been based on detection of viral RNA using reverse transcription polymerase chain reaction (RT-PCR). Detection rates can be affected by collection and storage of the specimen with varying results reported depending on testing of saliva, nasal, nasopharyngeal or rectal specimens [8–12]. Therefore, detection of antibodies against SARS-CoV-2 (IgM or IgG) in serum is likely to provide a more accurate estimation of the cumulative prevalence of SARS-CoV-2 in a population and as the vaccination schedule progresses, an understanding of the pregnant population response.

Our study aimed to understand the antibody response to SARS-CoV-2 in a cohort of symptomatic and asymptomatic pregnant women. We assessed SARS-CoV-2 in pregnancy with combination of RT-PCR and, using three independent assays, serological detection of anti-SARSCoV-2 antibodies. In addition, we obtained umbilical cord blood samples to matched RT-PCR positive or serological positive mothers and therefore we also present evidence of transplacental passage of anti-SARS-CoV-2 antibodies.

## Materials and methods

### Study design

This is a multicentre prospective observational study, conducted between the 23rd March and the 31st August, at two free-standing tertiary level university maternity hospitals in Dublin, Ireland. The Rotunda Hospital and The National Maternity Hospital provide both routine obstetric care and complex tertiary referral care for the city of Dublin and their national referral catchment areas. Each hospital delivers over 8,000 babies per annum and are amongst the largest maternity hospitals in Europe. Together, approximately 28% of the population of Ireland is delivered in the Rotunda and National Maternity Hospital annually. The study was approved by the ethics committee of both institutions. Informed written consent was obtained from all participants.

#### Cohort 1 symptomatic pregnant women

Twenty three consecutive women who were symptomatic of SARS-CoV-2 including fever, cough, shortness of breath and or anosmia, attended the hospital for a RT-PCR test between 24th March and 30st April 2020. Those that attended community testing centres and had a positive RT-PCR test were identified via the hospital infection control team. Within this whole cohort (n = 23), ten RT-PCR positive women were identified (10/23, 43%). A 5ml serum and 5ml EDTA sample were taken on the day of presenting symptoms, or, in the patients tested and diagnosed in the community, the serum was obtained when they attended the hospital outpatients at various time points during their convalescence. Serum analysis, was therefore performed on day of presenting symptoms or as far out as day 66 post initial symptoms. All samples were analysed for the presence of IgG and IgM anti-SARS-CoV-2 antibodies. Participants also consented to an umbilical cord sample on delivery.

#### Cohort 2 asymptomatic pregnant women

Following identification of the SARS-CoV-2 positive women, we proceeded to assess the prevalence in a large scale study of asymptomatic women, initiated from the 4th May to 15th May 2020. Eligible participants were identified from both inpatients and outpatient clinics. Patients were screened with a questionnaire for symptoms of SARS-CoV-2 at the time of the study and excluded if they had symptoms suggestive of active and or recent infection (within 14 days). These women were offered RT-PCR testing separate from the research study.

We approached 923 women and a total of 608 consented to and had a nasopharyngeal swab analysed for RT-PCR. Five hundred and ninety-eight of these women consented to provide a blood sample for immunological analysis. Samples collected from participants were processed immediately and stored at -80°C prior to analysis. Nine of the women who were positive for IgG or IgM returned for longitudinal assessment of antibody response on days 101–122 post baseline testing.

### Collection of cord blood

All participating women who delivered during the study period both in the symptomatic and asymptomatic cohort, were also asked for consent for a sample of umbilical cord blood at delivery. A 5ml serum and 5ml EDTA venous sample was taken from the cord after the baby was delivered and the cord was clamped. These were then processed as per the maternal serological samples.

### Processing and analysis of respiratory samples

Respiratory samples were inactivated by incubation with a lysis buffer containing guanidinium thiocyanate in a biological safety cabinet for 10 minutes prior to analysis. SARS-CoV-2 RNA testing was performed on the cobas SARS-CoV-2 6800 (Roche Molecular Systems, Branchburg, NJ) in accordance with the manufacturer’s instructions. A 0·6 mL aliquot of each sample was loaded onto the cobas 6800 where it was combined with the cobas SARS-CoV-2 master mix containing an internal RNA control primers, and probes targeting the ORF1/a nonstructural region that is specific for SARS-CoV-2 (target 1), as well as the conserved, structural protein envelope E gene that is shared by the Sarbecovirus subgenus (target 2). Results were reported by the cobas SARS-CoV-2 test as either “detected” (targets 1 and 2 detected), “presumptive positive” (target 1 not detected; target 2 detected), or “not detected”.

### Processing and analysis of serology

All serological samples were processed in a single laboratory (Core Laboratory in the Clinical Research Centre, University College Dublin), in a blinded fashion using three different assays on three different platforms: the electrochemiluminescence immunoassay [13] on an automated Roche platform Cobas® e411, and the chemiluminescent microparticle immunoassay (CMIA) (SARS-CoV-2 IgG 75 assay; Abbott Laboratories, IL, USA) on Architect i2000SR and Alinity and the chemiluminescent microparticle immunoassay (CMIA) (SARS-CoV-2 IgM assay; Abbott Laboratories, IL, USA) on Architect i2000SR Plasma samples were processed immediately after collection and stored at -80°C prior to analysis. The Elecsys anti-SARS-CoV-2 serology assay is a sandwich immunoassay intended for the detection of IgM and IgG antibodies to SARS-CoV-2 in human serum and plasma. 140 μL (20uL 9 +120uL dead volume) of sample was used in the assay. Results were determined automatically by the software by comparing the electrochemiluminescence signal obtained from the reaction product of the sample with the signal of the cut-off value previously obtained by calibration with ACOV2 Cal1 containing human serum, non-reactive for anti-SARS-CoV-2 antibodies, and ACOV Cal2 containing human serum reactive for anti-SARS-CoV-2 antibodies. Plasma samples were also run on the Abbott Architect i2000SR and the new Alinity instruments using the Abbott SARS-CoV-2 IgG assay. The assay is a two-step immunoassay using CMIA technology for qualitative detection of IgG in human serum or plasma, raised against the nucleocapsid protein of SARS-CoV-2. The Architect requires a volume of 75μL of serum or plasma (25uL+50uL dead volume). Qualitative results and index values reported by the instrument were used for analysis [14]. A signal/cut-off (S/CO) ratio of ≥1.4 was interpreted as reactive. Calibration was performed and positive quality control S/CO 1.65–8.40 and negative quality control S/CO ≤ 0.78 were fulfilled prior to analyses of patient samples. Within-day imprecision assessment was performed using QC material. IgM Plasma samples were run on the Abbott Architect i2000SR. A signal/cut-off (S/CO) ratio of ≥1.0 was interpreted as reactive.

### Statistical analyses

At the prevalence study (asymptomatic cohort) design stage, it was estimated that the asymptomatic prevalence of SARS-Co-V-2 would likely range between 15 and 20% [15, 16]. The sensitivity of current RT-PCR assays for SARS-CoV-2 has not been published but was expected to range from 70–90%. Specificity was expected to be above 90%. Therefore, to adequately power a study with a 10% prevalence rate would require between 355 and 574 patients, while if prevalence was found to be 20% this study would require between 462 and 733 10 patients. Median and interquartile range (IQR) are calculated for continuous variables, while counts and percentages are used to describe categorical variables. The Clopper-Pearson interval was used to find a 95% confidence interval for the probability of having positive seroprevalence results.

## Results

We established two cohorts of pregnant women in whom serological assessment of both IgG and IgM antibody response to SARS-CoV-2 was assessed. The demographic characteristics of the study population are demonstrated in S1 Table. Combining both cohorts, IgM anti SARS-CoV-2 antibodies were detected in 28 women, IgG antibodies were detected in 15 women and 13 women were positive for both IgG and IgM.

### Cohort 1

**Fig 1:**
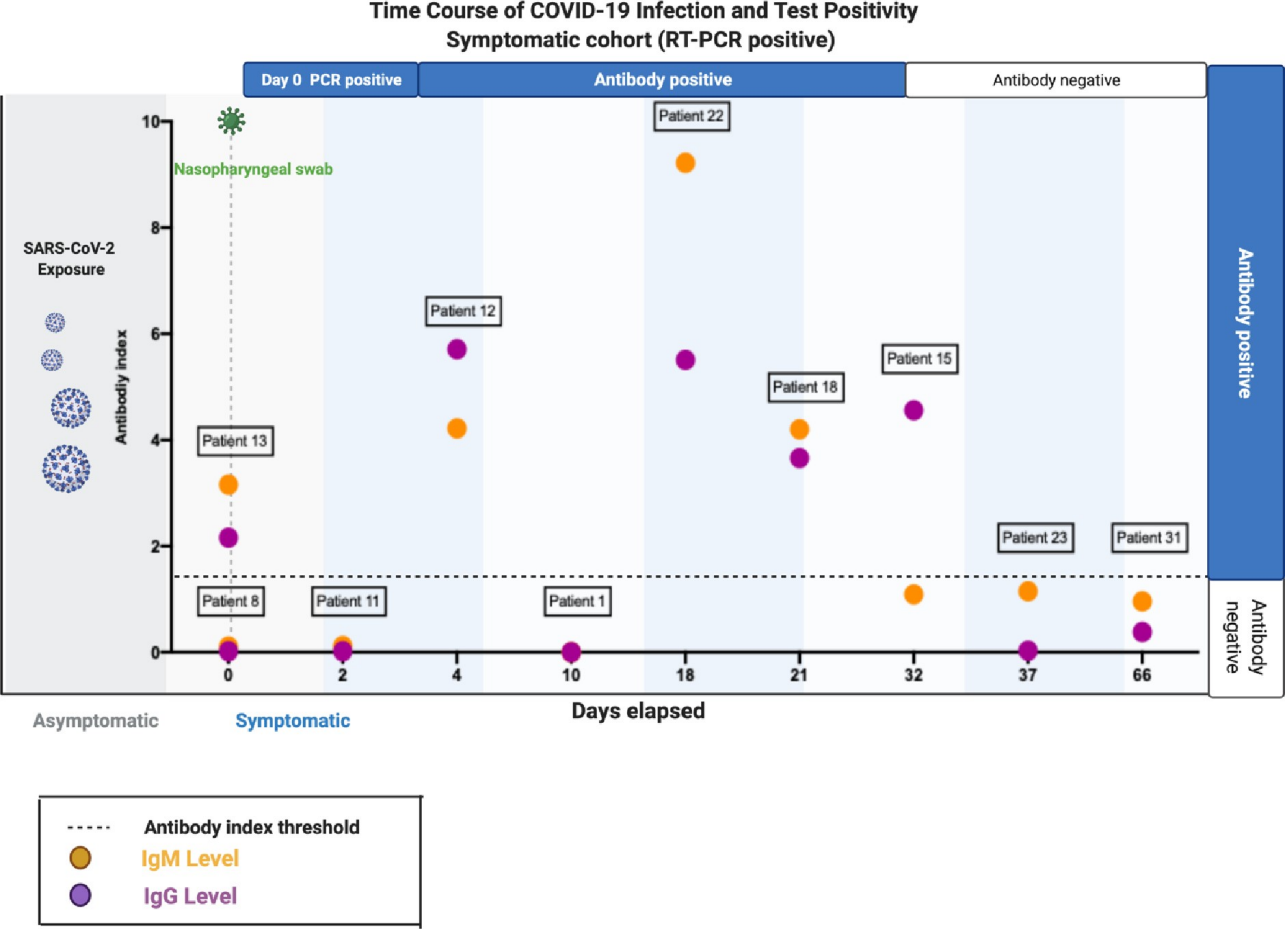
Anti-SARS-CoV-2 IgG and IgM antibody response in symptomatic pregnant women. Demonstration of IgG and IgM anti SARS-CoV-2 antibody response in RT-PCR positive patients. Days elapsed since RT-PCR range from 0–66 days with positive antibody response present from day 0–32.

Cohort 1 consists of 23 symptomatic women, ten of whom were confirmed to be SARS-CoV-2 infected via RT-PCR (S2 Table). After a positive RT-PCR test, IgG and IgM antibodies were measured at various points during convalescence. The mean time from positive RT-PCR to serological test was on day 21.8 days (range 0–66 days) (Fig 1). Anti-SARS-CoV-2 IgG antibodies were detected in 50% (5/10) of the RT-PCR positive SARS-CoV-2 women using the Roche and Abbot Architect platforms. Anti-SARS-CoV-2 IgM antibodies were detected in seven women in Cohort 1, one of whom was both RT-PCR negative on nasopharyngeal swab and IgG negative in serum which may reflect a false positive IgM. One woman, who was RT-PCR positive, did not mount an antibody response, when tested at day 10. She described very mild symptoms, presenting with an uncomplicated pyrexia and no respiratory symptoms.

The earliest evidence of a maternal IgG and IgM antibody response after a positive nasopharyngeal swab was at day 0. However, this patient reported first onset of symptoms 14 days prior to her nasal RT-PCR testing (Patient 13 Fig 1). Therefore, we determined that the that the earliest response between swab positive (at symptom onset) and a demonstrably IgG antibody response was at day 4 (Patient 12, Fig 1). Positive antibodies were detected in women tested between day 0 and day 32 after a positive RT-PCR. Detectable antibody levels were not present in women who were tested at day 37 and 66 after a positive nasopharyngeal swab.

### Cohort 2 RT PCR testing

Of 608 asymptomatic women who had a nasopharyngeal swab, SARS-CoV-2 RNA was detected in one woman, suggesting a prevalence of asymptomatic infection of 0·16% (1/608, 95%CI 0%- 0·9%). This lady tested positive for IgG SARS-CoV-2 antibodies but was negative for IgM antibodies. SARS-CoV-2 RNA was not detected in a nasopharyngeal swab from her baby. The umbilical cord blood, however, was positive for the presence of IgG anti-SARS-CoV-2 antibodies.

#### Cohort 2: Serological assessment in asymptomatic pregnant women

Samples were available for serological analysis in 598 women in the asymptomatic cohort. AntiSARS-CoV-2 IgG antibodies were detected in 12 women using the Roche platform and ten women using the Alinity and Architect platform. The seroprevalence rate using two independent assays was 1.67% (10/598 95% CI 0·8–3·1%). Anti-SARS-CoV-2 IgM antibodies were observed in twenty-one asymptomatic women (21/598 3.51% CI 2.3–5.5%). Of these 21 women, 8 were also IgG positive (8/21, 38%) using both the Roche, Architect and Alinity platforms. The majority of antibody positive women were greater than 24 weeks gestation (IgG n = 13, 84%, IgM n = 14, 50%). Demographics of all women are presented in S1 Table.

**Fig 2:**
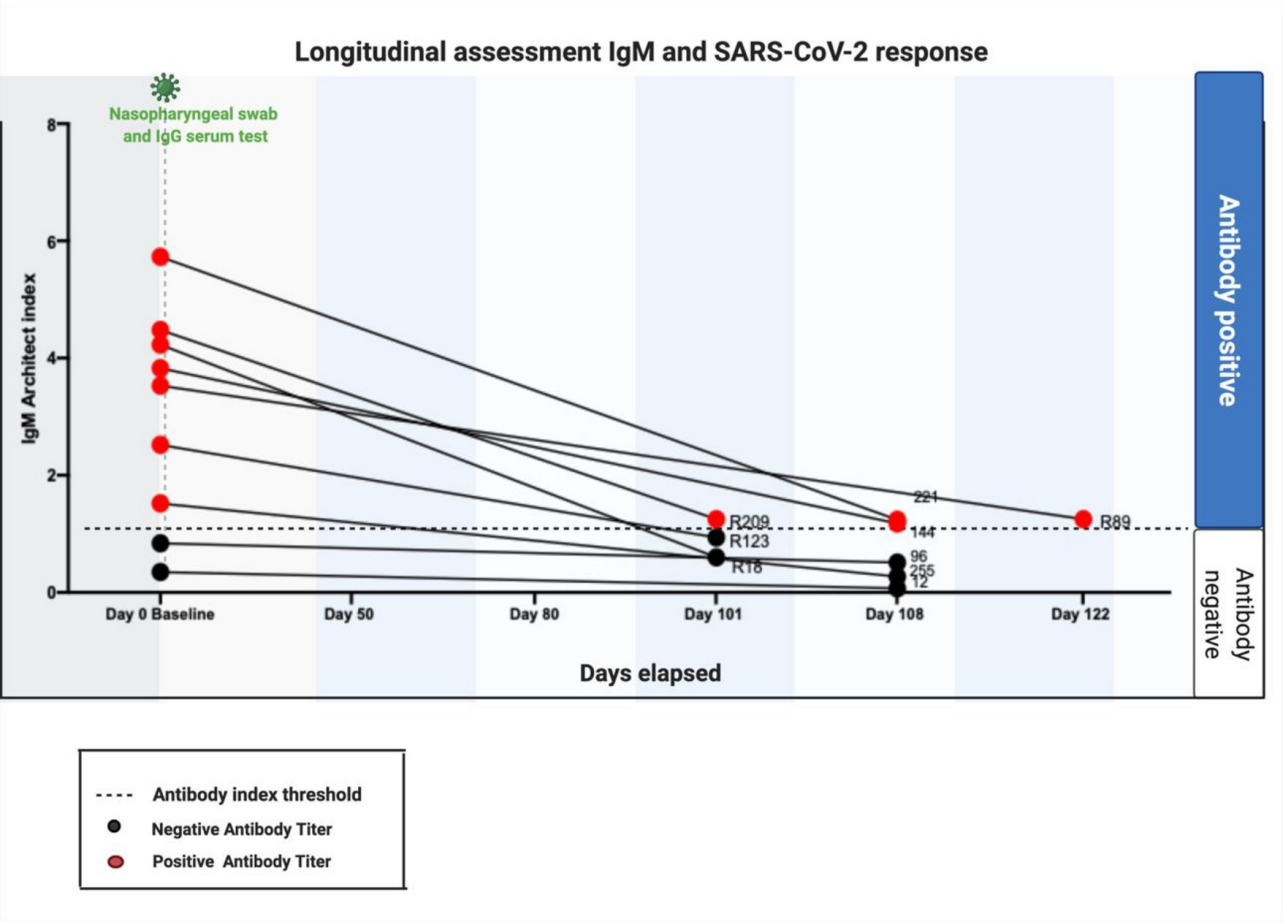
Longitudinal assessment of anti-SARS-CoV-2 IgM and IgG in pregnancy and the puerperium. IgM Baseline and Follow up results. Four women (4/9, 44%) remained IgM positive when tested for anti-SARS-CoV-2 on follow up.

**Fig 3:**
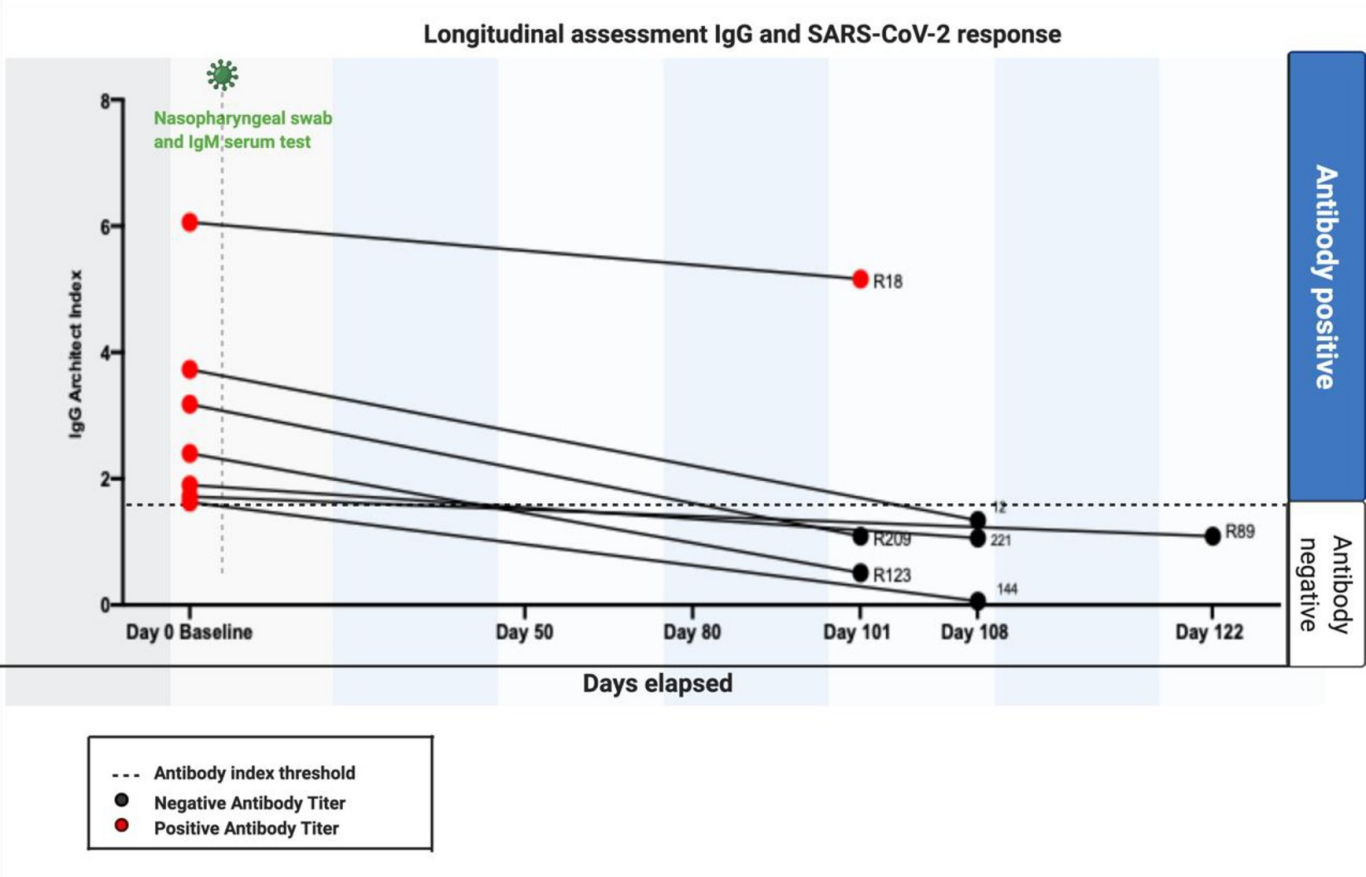
IgG baseline and follow up results. IgG Baseline and Follow up results. The majority (6/7, 85.7%) of the seven women who were IgG positive at baseline no longer exhibited anti SARSCoV-2 IgG antibodies.

Antibody positive women were invited to attend for a follow up serum analysis as part of this study. Nine women from the asymptomatic cohort returned for follow up testing ranging between 101–122 days after the baseline test (Table 1). Four (4/9, 44%) remained IgM positive (Fig 2). One woman was IgM positive only at baseline testing. Her follow up IgM antibodies were negative. In view of this, and the initial negative PCR and IgG anti SARS-CoV-2, this may represent a false positive IgM result, or, alternatively, this individuals IgM response had waned by the time of re-test. The majority (6/7, 85.7%) of the seven women who were IgG positive at baseline no longer exhibited anti SARS-CoV-2 IgG antibodies (Fig 3). Retrospective histories taken at follow-up suggests many of these women may have been mildly symptomatic at the time of the initial testing. The one lady who remained IgG positive had a history of asthma and a raised BMI (28 mg/kg2), who required hospital admission for observation.

**Table 1:** Follow up serology IgM and IgG (asymptomatic cohort).

No	Patient ID	Age	Gestation at Baseline testing	Baseline	Baseline	Follow up IgM Architect	Follow up interpretation	Day at follow up	Baseline IgG Architect	Baseline	Follow up IgG Architect	Follow up IgG Interpretation
										IgG Architect Interpretation		
				IgM Architect Index	IgM Interpretation							
								Serology				
1	R89	36	36	3.53	Positive	**1.25**	**Positive**	122	1.72	Positive	1.09	Negative
2	R123	34	34	2.52	Positive	0.94	Negative	101	2.40	Positive	0.51	Negative
3	R18	38	Postnatal	4.23	Positive	0.60	Negative	101	6.06	Positive	**5.16**	**Positive**
4	R209	35	37	4.48	Positive	**1.25**	**Positive**	101	3.18	Positive	1.09	Negative
5	12	40	34	0.35	Negative	0.07	Negative	108	3.73	Positive	1.34	Negative
6	144	37	41	5.73	Positive	**1.24**	**Positive**	108	1.63	Positive	0.60	Negative
7	96	22	29	0.84	Negative	0.51	Negative	108	1.16	Negative	0.28	Negative
8	221	29	32	3.83	Positive	**1.18**	**Positive**	108	1.90	Positive	1.06	Negative
9	255	37	28	1.52	Positive	0.27	Negative	108	0.83	Negative	0.27	Negative

Nine asymptomatic patients returned for follow up bloods between day 108–115 days post Baseline bloods

IgG: One of the seven IgG positive (Architect platform) women (1/7, 14.2%) remained IgG positive on follow up

IgM: Four of the nine IgM positive women (4/9, 44%) remained IgM positive on follow up

### Umbilical cord blood analysis

During the study period, seventy-eight women who participated in the study delivered their baby. Umbilical cord bloods (n = 78) were assessed for the presence of SARS-CoV-2 antibodies. Anti-SARS-CoV-2 IgG antibodies were detected in 5 umbilical cord blood samples (Table 2). IgM antibodies were not detected in any cord blood samples. All five of the corresponding maternal samples were seropositive during pregnancy. Four of these women also had a positive RT-PCR for SARS-CoV-2, of which one was asymptomatic of SARS-CoV-2. The mean time between serologic positivity and detection in umbilical cord samples was 28 days (range 0–66 days). The mean gestation at diagnosis of SARS-CoV-2 was 34 weeks, mean delivery gestation was 38 weeks. The median antibody index was 4.88 (Roche) and 2.33 using the Alinity platform.

**Table 2:** Umbilical cord bloods samples positive for SARS-Co-V2.

Patient ID	Gestation at maternal RT-PCR swab	Result of maternal RT PCR test	Gestation at Delivery	Umbilical cord blood	Umbilical cord blood	Umbilical cord blood	Umbilical cord blood	Umbilical cord blood	Umbilical cord blood
								IgM Architect Assay	IgM Interpretation
				Roche COI result	Roche interpretation	IgG Alinity	Alinity Interpretation		
Patient 10	30	Positive	38	26.14	Positive	3.52	Positive	0.01	Negative
Patient 12*	34	Positive	34	2.66	Positive	2.23	Positive	0.01	Negative
Patient 13	36	Positive	39	21.51	Positive	3.59	Positive	0.03	Negative
Patient 31	31	Positive	40	3.31	Positive	1.44	Positive	0.01	Negative
Patient 144*	41	negative	41	4.88	Positive	2.21	Positive	0.02	Negative

* Patient asymptomatic of SARS-CoV-2.

Positive umbilical cord bloods: RT-PCR Swab and antibody results Positive umbilical cord bloods: RT-PCR Swab and antibody results.

## Discussion

### Main findings

We present a comprehensive profile of the antibody responses to SARS-CoV-2 in both symptomatic and asymptomatic pregnant women. Anti-SARS-CoV-2 antibodies can be detected in symptomatic pregnant women at various stages of convalescence from the virus, we characterise the positive antibody response for up to 32 days after a positive RT-PCR test (Fig 1). We also present longitudinal analysis revealing key temporal features of the antibody response to SARS-CoV-2 in pregnancy and the early post-natal period, providing important additional information regarding disease trajectory in pregnancy. A longitudinal study of asymptomatic or mildly symptomatic healthcare workers in Belgium demonstrated 91% had detectable IgG antibodies at 120 days after infection [17]. This initial analysis suggests that in our cohort, antibody response in pregnant women was less durable than in the non-pregnant population although further population studies are required and information gathered following widespread vaccination programmes, will add to our knowledge regarding the antibody response in pregnancy.

Reassuringly, our finding of anti-SARS-CoV-2 IgG antibodies in umbilical cord blood raises the possibility that passive immunity was established in babies born to mothers with a history of SARS-CoV-2 infection. Both of these findings are likely to have an impact on vaccination strategies and highlight the importance in confirming the safety and efficacy of any vaccine in the pregnant population.

#### Study strengths

One of the major strengths of our study is the use of two independent assays to confirm seroprevalence and antibody levels. The Elecsys Anti-SARS-CoV-2 assay uses a recombinant protein representing the nucleocapsid (N) antigen for the determination of antibodies against SARS-CoV-2 [13]. The Abbot SARS-CoV-2 IgG assay is a chemiluminescent microparticle 17 immunoassay (CMIA) intended for the qualitative detection of IgG antibodies to SARS-CoV-2, which also targets the nucleocapsid protein. Both tests enable a comprehensive determination of the immune reaction to SARS-CoV-2 and provides reliable sensitivity and specificity [13]. The accuracy could be further strengthened by using assays directed against anti receptor binding domain (RBD), which have been shown to persist for longer periods [18]. Using assays directed to the anti RBD have increased detection of anti-SARS-CoV-2 antibodies above the use of Abbot and Roche systems alone [19]. As a result, the seroprevalence may be underestimated in this cohort. In addition, testing for IgA could further validate the study findings. IgA can also remain elevated for up to 50 to 60 days after the onset of symptoms [9].

Our demonstration of anti- SARS-CoV-2 IgG antibodies in five umbilical cord samples taken in women who had confirmed evidence of infection, is noteworthy and confirms transplacental passage of anti-SARS-CoV-2 antibodies and raises the possibility of the presence of passive immunity. As the pandemic has progressed, transplacental pass of SARS-CoV-2 RNA has been demonstrated in the literature and further evidence is beginning to emerge of the SARS-CoV-2 antibodies within umbilical cord [7]. Our confirmation of transplacental passage of IgG antibodies could potentially play an important role in ongoing vaccination strategies. These findings are particularly important as a numerous groups have advocated for vaccination of pregnancy women as an at-risk groups [20]. These data may therefore help direct antenatal vaccination programs. Antibodies have been detected in cord bloods of up to 80% of babies born to mothers who participated in an RSV vaccination schedule [21]. 18 In addition to confirming the presence of antibodies in umbilical cord blood, we provide a first assessment of natural history of antibodies in the fetal system after transplacental migration. We detected IgG antibodies in an umbilical cord blood sample 66 days after diagnosis of maternal SARS-CoV-2 suggesting transplacental passage and persistence of antibodies even when maternal antibodies have waned. While anti-SARS-CoV-2 antibodies were no longer detectable in maternal blood sample, the cord blood was positive for IgG anti SARS-CoV-2. (Patient 31, Table 2). Transplacental migration of IgG antibodies begins from 13 weeks and peak in the second and third trimester [22], clearly demonstrated by studies of antenatal influenza vaccination where cord blood antibody levels are significantly higher when the mother is vaccinated in either trimesters 2 or 3 [23, 24] The efficiency of IgG transfer can vary from one antigen-specificity to another. In normal pregnancy, the transfer efficiency of IgG against pertussis can be up to 200% whereas for group B streptococcus it is only 70% [25]. Our findings of IgG in umbilical cord blood born suggest efficiency of transfer of IgG in the novel SARS-CoV-2. However, the timing of infection may influence transfer as the SARS-CoV-2 specific antibody transfer has been noted to be reduced when compared to influenza and pertussis in the third trimester only.

Larger cohorts will be required to substantiate our findings.

Serological assessment of pregnant women may provide a more accurate assessment of seroprevalence. A study from Philadelphia demonstrated a seropositivity rate of 6.2% (80/1293) in a pregnant population. This was considerably higher than the estimated infection rate of 1.4% in that areas general population [26]. We detected a very low prevalence rate from RT-PCR alone (1/608, 0·16%, 95%CI 0%-0·9%). However, in keeping with other published reports, our study of asymptomatic women also demonstrates a very low serological prevalence SARS-CoV2 [27]. Whilst the seropositivity rate of IgM was almost three times higher than that of IgG anti-SARS-CoV-2 antibodies (IgM = 21/598, 3.51% and IgG = 10/598, 1.67%) both were low within our asymptomatic population.

Our assessment of the temporal serological response in pregnancy suggests that IgM antibodies persisted for over 100 days in four of nine patients (Fig 2) while persistent IgG positivity was only seen in one patient (Fig 3). In other longitudinal studies of antibodies in a non-pregnant populations, IgM levels decreased rapidly in recovered patients [28]. Our pregnant population were asymptomatic or exhibited mild symptoms only. In previous SARS pandemics, IgM antibodies to SARS‐CoV persist for a much shorter period of time and detectable IgG antibodies and neutralizing viral antibodies persisted for up to 720 days [26, 28]. Longitudinal assessment of antibody response to other viral infections in pregnancy have shown that CMV IgM has been shown to peak during the first 1 to 3 months after primary infection in pregnant women and then persist at a low level for 18 to 39 weeks [28]. The zika virus specific IgG/IgM antibody has also been demonstrated to be sustained throughout pregnancy and postpartum [29] In SARS-CoV-1 infected patients, 90% and 50% have been shown to maintain IgG antibodies for two and three years respectively [3]. The transfer of SARS-CoV-2 is impacted by the timing of infection and is compromised in the third trimester. The virus does not alter the antibody glycome and changes in placental Fc receptors may impact the transfer of antibodies in the third trimester. Taken together, our data suggest that SARS-CoV-2 exhibits a different phenotype and clinical course than other viral infections in pregnancy. Larger cohort studies are needed to validate these findings.

#### Study limitation

The asymptomatic prevalence study was implemented nine weeks after the first confirmed case of SARS-CoV-2 infection was reported in Ireland (March 1st 2020) and 16 days after the peak of the 20 population infection [27]. Our study commenced as the rate of new cases of infection was already falling and therefore may account, in part, for the low prevalence of PCR positive cases in this cohort. However, the low antibody prevalence in our study correlates with other large population based studies and suggests that even at this stage of the pandemic, the vast majority of the pregnant population remains immunologically naïve. Only five seropositive patients delivered during this study period. Further analysis of the transplacental passage of antibodies in umbilical cord blood in larger cohorts, will provide further credence to our findings and is required to assist in counselling pregnant women and directing future vaccination strategies. As previously explained, inclusion of assays directed against anti RBD antigen, in addition to detection of antiSARS-CoV-2 IgA antibodies, could also increase the detection of seropositivity and thereby strengthen the validity of this study [18].

## Conclusion

Large scale and comprehensive assessment of the IgG and IgM antibody response to SARS-CoV2 is vital to determine the aetiology of the virus within the pregnant population. Further analysis can confirm the transplacental transmission of anti-SARS-CoV-2 antibodies and characterize the maternal temporal response. This information could inform future public health policy regarding antenatal immunisation programs and neonatal care.

## Supporting information

S1 Fig Consort diagram of asymptomatic prevalence of SARS-CoV-2 infection in pregnancy. (TIF)

S1 Table Asymptomatic and symptomatic cohort: All patient demographics. (TIF)

S2 Table Symptomatic cohort. Table of confirmed RT-PCR positive for SARS-Co-V2 on nasopharyngeal swab (n = 10) with corresponding serological antibody results.
(TIF)

S3 Table Case report form asymptomatic cohort. (TIF)
