# Supplementary figures and images for: Immunological assessment of SARS-CoV-2 infection in pregnancy from diagnosis to delivery: A multicentre prospective study
Source: PLoS One. 2021 Sep 20;16(9):e0253090. doi: 10.1371/journal.pone.0253090 (PMC8451988; doi:10.1371/journal.pone.0253090)

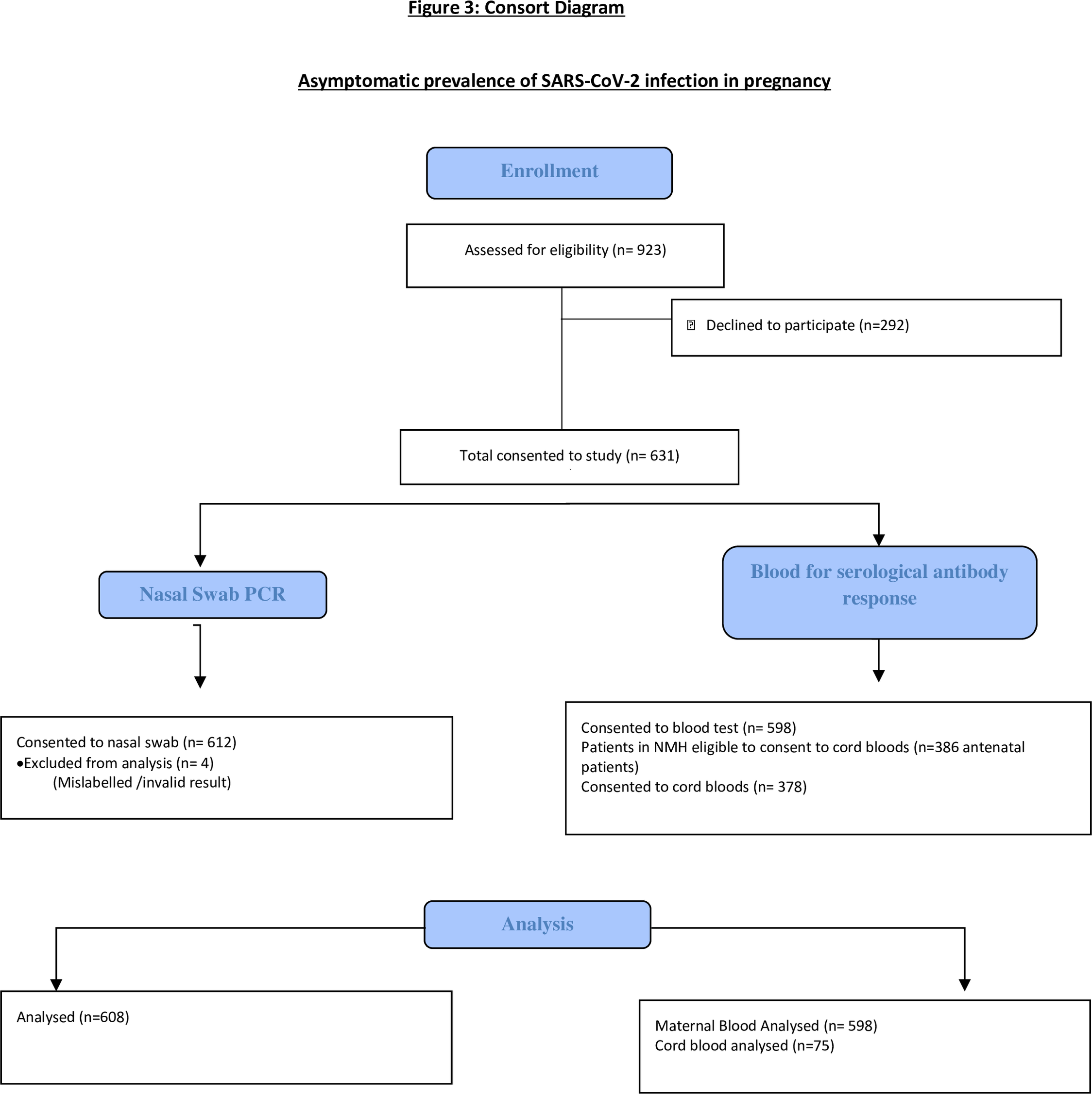

Supplement: S1 Fig — (TIF) [file pone.0253090.s001.tif]

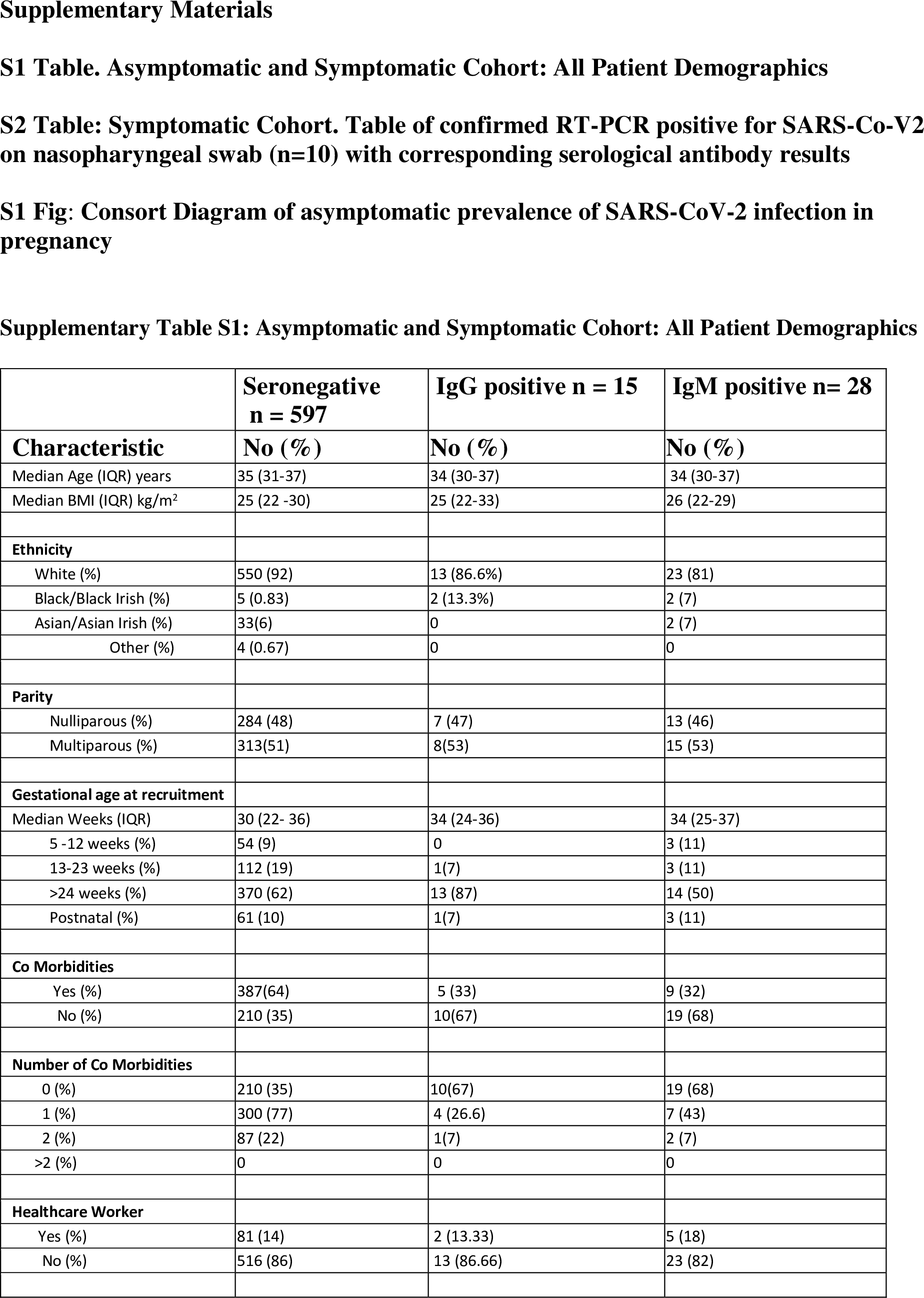

Supplement: S1 Table — (TIF) [file pone.0253090.s002.tif]

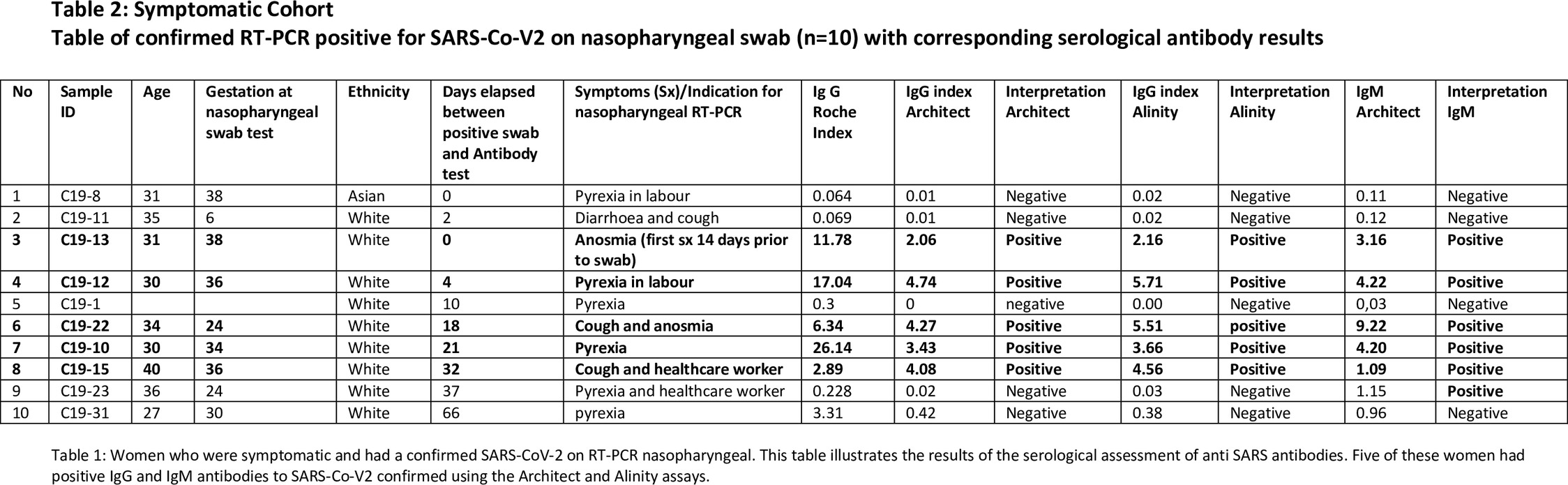

Supplement: S2 Table — Table of confirmed RT-PCR positive for SARS-Co-V2 on nasopharyngeal swab (n = 10) with corresponding serological antibody results. (TIF) [file pone.0253090.s003.tif]

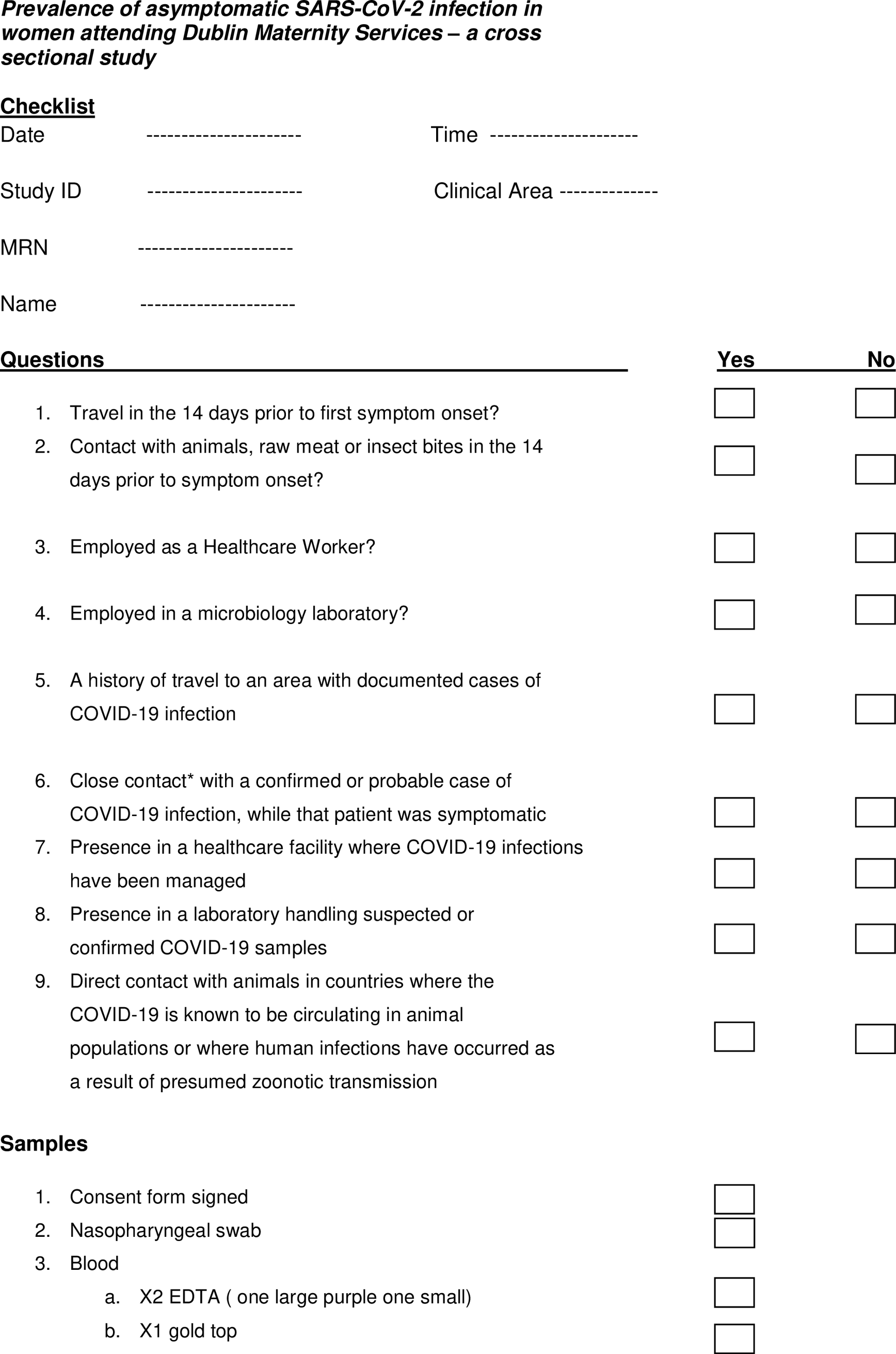

Supplement: S3 Table — (TIF) [file pone.0253090.s004.tif]
